# Ameliorative effects of silymarin on HCl-induced acute lung injury in rats; role of the Nrf-2/HO-1 pathway

**DOI:** 10.22038/IJBMS.2019.14069

**Published:** 2019-12

**Authors:** Rasha F Ahmed, Rabab A Moussa, Reda S Eldemerdash, Mahmoud M Zakaria, Seham A Abdel-Gaber

**Affiliations:** 1 Department of Medical Biochemistry, Faculty of Medicine, Minia University, 61511 Minia, Egypt.; 2 Department of Pathology, Faculty of Medicine, Minia University, 61511 Minia, Egypt.; 3 Research Building, Urology & Nephrology Center, Mansoura University, 35516 Mansoura, Egypt.; 4 Department of Pharmacology, Faculty of Medicine, Minia University, 61511 Minia, Egypt

**Keywords:** Acute lung injury, Fibrosis, Heme oxygenase-1, Silymarin, Survivin

## Abstract

**Objective(s):**

Aspiration is a common cause of acute lung injury (ALI), which lacks an effective treatment. Inflammation and oxidative stress play key roles in ALI development. Silymarin is an active extract of Silybum marianum plant seeds (milk thistle). Silymarin has potent anti-inflammatory and antioxidant effects; however its role in aspiration induced ALI has not been investigated. The aim of this study is to investigate the role of silymarin in the treatment of hydrochloric acid (HCl) aspiration induced ALI and explores its mechanisms of action.

**Materials and Methods:**

The study included three groups of rats: Control non-treated group, ALI group (intra-tracheal HCl injected), and silymarin treated ALI group. White blood cells (WBCs) with differential count, oxidative stress parameters, B-cell lymphoma 2 (Bcl-2), transforming growth factor-beta (TGF-β), cyclooxygenase 2 (COX-2), nuclear factor erythroid 2-related factor-2 (Nrf-2), and heme oxygenase-1 (HO-1) were investigated. Lung tissue histopathology and immunohistochemical expression of survivin and proliferating cell nuclear antigen (PCNA) were also examined.

**Results:**

The results of the study showed that HCL caused histopathological changes in ALI with leukocytopenia and increased oxidative stress biomarkers. It increased TGF-β, up-regulated mRNA expression of COX-2, Nrf-2, and HO-1 and increased survivin and PCNA but decreased Bcl-2. Silymarin ameliorated the histopathological lung injury with further up-regulation of Nrf-2 and HO-1 mRNA and decreased the inflammatory and fibrotic parameters together with up-regulation of the anti-apoptotic and the proliferation parameters.

**Conclusion:**

The protective effect of silymarin against ALI is mediated by Nrf-2/HO-1 pathway with subsequent antioxidant, anti-inflammatory, antiapoptotic, and proliferating activities.

## Introduction

Acute lung injury (ALI) refers to acute, persistent lung inflammation accompanied by increased vascular permeability leading to over-activation of macrophages and neutrophils, excessive release of inflammation-associated proteases and reactive oxygen species (ROS) and pulmonary hemorrhage (1). A variety of exogenous and endogenous factors can lead to ALI due to diffuse alveolar damage and acute respiratory distress syndrome (ARDS) where the inflammation must be sufficiently severe and lead to hypoxemia (2, 3). The most common causes of ALI/ARDS disease include sepsis, trauma, aspiration, multiple blood transfusions, acute pancreatitis, inhalation injury, and certain types of drug toxicity (4). The pathological features of ARDs include rapid recruitment of WBCs and release of pro-inflammatory cytokines, triggering systemic inflammation and fibro-proliferative phase (4).

Gastric content aspiration is an important risk factor for ARDS. HCl is the main factor of injury in gastric content and can induce lung injury in the animal model (5). Usually, bleomycin or paraquat are standard models of inducing lung injury in animals, and use of HCl is not usual (6, 7), however gastric acid aspiration induced lung injury carries great importance for many reasons. Firstly, there is a broad range of conditions that predispose to gastric aspiration–induced ALI including general anesthesia, alcohol and narcotic abuse, and neurologic disorders. Secondly, gastric acid aspiration results in a spectrum of potential outcomes ranging from being asymptomatic, producing a rapidly resolved pneumonitis, or progression to a severe and sustained ALI. ARDS is seen in 10% to 25% of witnessed gastric aspiration events and carries a mortality rate of 35% to 60% (8).

Acid aspiration is known as a neutrophil-dependent form of ALI characterized by injury of both capillary endothelium and alveolar epithelium. The initial chemical damage to pulmonary airway epithelium is exerted by HCl, which triggers an inflammatory response followed by pulmonary edema and disruption of the alveolar-capillary membrane (9). Most of the animal models of ARDS show a spontaneous recovery of lung injury in 24 or 48 hr (10).

Nuclear factor erythroid 2-related factor 2 (Nrf-2) is a transcription factor, which is widely expressed in many organs and is considered a multiorgan protector (11). Nrf-2 regulates expression of many genes encoding antioxidant activities, including heme oxygenase 1 (HO-1) (12)**.**

Silymarin is the active principle of *Silybum marianum* seeds (milk thistle) constituting about 80% of all seed extracts and has potent anti-oxidant, anti-inﬂammatory, and anti-carcinogenic properties (13, 14). Moreover, it has been found to protect many organs including the lungs (7), liver (15), and mesentery (16) against injury caused by different etiologies. The protective effect of silymarin in a variety of diseases is carried out by inhibiting secretion of certain pro-inflammatory cytokines and impairing neutrophils migration (17). The combination strategy of both anti-inflammation and anti-oxidant activities is a good choice for ARDS prevention and treatment (18). Silymarin was previously evaluated to be protective in different models of lung injury including lipopolysaccharide (LPS) (18) induced lung injury with proven anti-inflammatory activity, however this study lacks evaluation of the molecular mechanism of this anti-inflammatory activity. It was also evaluated in paraquat-induced (7) lung injury with evaluation of Nrf-2 as a molecular mechanism, however this effect was evaluated in the early stages of lung injury.

The antioxidant defense mechanisms of silymarin were evidenced in different tissues, including lung, and were detected to exert their antioxidant effect via five different mechanisms including down-regulation of inflammatory markers through Nrf-2 mediated mechanism (13).

Despite the anti-oxidant and anti-inflammatory activities of silymarin, its role has not been investigated in HCl induced ALI and not evaluated in the late stages of lung injury by any model. Therefore, the aim of the current study is to evaluate the possible protective effects of silymarin in HCl aspiration induced ALI and evaluating its mechanism of action and its relation to Nrf-2/HO-1. 

## Materials and Methods

***Animals***

Thirty adult male inbred Sprague-Dawley rats (about three months old), initially weighing an average of 180–200 g, were obtained from the Nile Center of Experimental Research, Mansoura, Egypt. Then animals were housed in stainless steel cages in an artificially illuminated and thermally controlled room (22–25 °C and 12 hr light /dark cycle). Rats were fed normal laboratory rodent diet and given water *ad libitum *for one week of acclimation prior to the experimental work. All experiments were conducted in adherence to the ethical standards approved by the faculty board committee of the Faculty of Medicine, Minia University, Egypt. Animals received care in accordance with the ethics included in the Declaration of Helsinki for animal experiments**.**

***Chemicals and antibodies***

Silymarin (Sigma-Aldrich, USA), superoxide dismutase (SOD), catalase (CAT), and malondialdehyde (MDA) kits were purchased from (Bio Diagnostic, Egypt). Anti-survivin and anti-proliferating cell nuclear antigen (PCNA) and rabbit polyclonal antibodies were purchased from Thermo Fisher Scientific Inc/Lab Vision (Fremont, CA, USA). All other used chemicals were obtained from their commercial sources.

***Groups and experimental design***

Rats were divided into three experimental groups. Rats in group 1 (n=10) served as normal control injected with normal saline into their lungs and orally treated with the silymarin vehicle. Group 2 (ALI group; n=10) injected with HCl (0.1 N, pH 1.25) into the lungs in a volume of 1.2 ml/kg and orally treated with the vehicle (19). Group 3 (n=10) injected with HCl and treated by silymarin suspended in 0.5% carboxymethyl cellulose in a dose of 200 mg/kg orally for seven days (20). For injection of solutions, rats were anesthetized via ketamine/xylazine (100/10 mg/kg) (21) and then placed in a supine position with the extremities pull caudally to help exposure of the trachea. After that, the trachea was exposed through an anterior neck incision, and a direct puncture with a 24-gauge needle on a 1 ml tuberculin syringe was performed at two to four tracheal rings below the larynx. Saline or HCl was injected into the lung in a volume of 1.2 ml/kg; then the tuberculin syringe was removed. Finally, the neck was repaired with sutures. After seven days, animals were anesthetized, and lungs of each animal were surgically removed, then rinsed with normal saline and a tissue sample from each lung was taken for further investigations. Venous blood was collected from the jugular vein and centrifuged at 5000 rpm for 15 min (JanetzkiT30 centrifuge, Germany) for WBC count.

***WBCs, neutrophil, and lymphocyte count***

A sample of whole blood was placed in an EDTA-added vial to generate a complete blood count with cellular differential, before its measurement by an automated system. 

***Estimation of oxidative stress parameters***

Lung tissue specimen from each rat was immersed in liquid nitrogen just after scarification then kept in -80 °C for estimation of SOD, CAT activities, and MDA level. The activities of SOD and CAT in lung tissues were measured kinetically using a commercially available kit according to the manufacturer’s instructions (Bio Diagnostic, Egypt). The results were calculated and expressed as U/g tissue. Lung tissue content of MDA was estimated spectrophotometrically using a commercial kit according to the manufacturer’s instructions (Bio Diagnostic, Egypt). Results of MDA were expressed as nmol/g tissue. 

***Flow cytometric analysis***

B-cell lymphoma 2 (Bcl-2) and transforming growth factor-beta (TGF-β) were detected using flow cytometric analysis. Fresh lung tissues from all groups were cut into small pieces in Dulbecco’s modified Eagle’s medium (DMEM, GIBCO) containing 20% fetal calf serum (FCS, GIBCO), 1% penicillin-streptomycin (PS, GIBCO), and collagenases I, II, and IV (1 mg/mL, Sigma), were then incubated at 37 °C for 1 hr on a thermal shaker. The final cell dissociation was achieved between ground glass slides. After that, cells were separated from pellets by centrifugation (2000 rpm, 10 min). Pellets were resuspended in erythrocyte lysis buffer (155 mM NH_4_Cl, 10 mM KHCO_3_, 0.1 mM EDTA) and incubated for 10 min at room temperature. 

Detection of Bcl-2 TGF-β for the lung cells, including control group was tested by fixing in 80% methanol for 5 min. Then, the cells were permeabilized with 0.1% PBS-Tween for 20 min. Thereafter, the cells were incubated in one fold PBS/10% normal goat serum/0.3 mol/L glycine to block non-specific protein-protein interactions followed by Anti Bcl-2A1 antibody (ab33862) diluted 1/100 (Abcam San Francisco, USA) for 30 min at room temperature and Anti-TGF-β antibody (354039) diluted 1/100 (BD Life Science, USA) for 30 min at room temperature.

***Real-time PCR***

Total RNA was extracted from the lung tissue of experimental rats just after removal from animals freshly by using the RNeasy Plus Mini kit (Qiagen GmbH, Hilden, Germany) which included a specially designed genomic eliminator spin column to remove DNA contamination, and according to manufacturer’s instruction the RNA was isolated. RNA samples which yielded 2 distinctive bands in agarose gel electrophoresis and their concentrations were measured by a spectrophotometer (Nanodrop 2000, Thermo Scientific, USA) in which 260/280 more than 2 and 260/230 more than 1.8 were intact and stored for cDNA conversion in liquid nitrogen. 2 µg of total RNA of each specimen was converted into cDNA using RT First Strand kit (Qiagen Sciences, Maryland, USA). Gene expression was examined for nuclear factor erythroid 2-like 2 (Nrf-2), heme oxygenase-1 (HO-1), and cyclooxygenase 2 (COX-2). Glyceraldehyde-3-phosphate dehydrogenase (GAPDH) was included as an internal control and for normalization. The primers were designed online at the NCBI website using Primer3 home page (Table 1). Amplifications were performed in 25 µl reaction volume in each tube, which contained 12.5 µl SYBR Green (SensiFast SYBR, Bioline, UK), 1 µl of cDNA template, 2 µl of 10 pM primers and 9.5 µl of nuclease-free water. Cycling protocol of PCR amplification was done as follows: initial denaturation at 95 °C for 2 min, followed by 40 cycles of amplifications (denaturation at 94 °C for 20 sec, annealing and extension at 60 °C for 1 min). For each sample, the procedure was carried out in triplicate. A mathematical model introduced by Pfaffl was used for the relative quantification of target genes (Pfaffl, 2001). Gene expression was expressed relative to that of the control group (Group 1).

***Histopathological examination***

The standard histological methods for processing the lung tissue include fixation in 10% buffered formalin (24 hr), embedding in paraffin, sectioning through five µm thick paraffin sections, and routine staining with hematoxylin and eosin (H&E) dye. The stained slides were microscopically analyzed using light microscopy (Olympus, Japan). A blinded pathological assessment was done to lung tissue samples of each group. The parameters of lung injury were congestion, edema, infiltration of inflammatory cells, and hemorrhage. Damage to the lung tissue was graded by a pathologist on a scale of 1 to 4 (1= absent and appears normal; 2= mild; 3= moderate; 4=severe) according to combined assessments of alveolar congestion, hemorrhage and edema, infiltration/aggregation of neutrophils in the air- space or vessel wall, and thickness of the alveolar wall (22) .

***Immunohistochemical examination***

Survivin and PCNA were immunohistochemically investigated. For immunohistochemical staining, Streptavidin-biotin immunoperoxidase complex procedure was applied. 5-μm-thick sections were transferred to adhesive slides from representative formalin-fixed, paraffin-embedded blocks. The sections were de-paraffinized in xylene and dehydrated through a series of graded alcohols. Endogenous peroxidase activity was blocked by incubation with 0.3% hydrogen peroxide in methanol for 30 min. Antigen retrieval was done by microwave treatment in sodium citrate buffer, pH 6, for both survivin and PCNA for 15 min. Tissue sections were then incubated with antibodies for survivin; rabbit polyclonal antibody, from (Thermo Fisher Scientific) was used at 1:50 dilution. Anti-PCNA, from (Santa Cruz), was used at 1:30 dilution for 30 min, followed by biotinylated secondary antibody for 30 min at room temperature. Visualization of the reaction was done with an avidin-biotin complex immunoperoxidase system using 3,3′ diaminobenzidine (DAB) as a chromogen. Sections were then counterstained with hematoxylin, dehydrated, cleared, and mounted with distyrene, plasticizer, and xylene (DPX). 

***Evaluation of immunostaining***

Both nuclear and cytoplasmic staining were positive for survivin. The survivin immunostaining was semi-quantitatively scored using the H-score, which was determined by adding the results of multiplication of the percentage of cells with staining intensity ordinal value (scored from 0 for “no signal” to 3 for “strong signal”) with 300 possible values. Positive cells below 1% was considered a negative result. Specimens with score 1 to 100, 101 to 200, and 201 to 300 were respectively classified as having low, intermediate, and high-level expression. 

As regards PCNA, nuclear staining is considered positive. Immunostaining was evaluated using Labeling Index (LI), which is determined by calculating the percentage of immunostained cells divided by the total number of cells in the evaluated area. From each specimen, 1000 cells were counted.

***Statistical analysis***

Statistical analysis was done using GraphPad Prism software (ver. 6). Result data were analyzed using one way ANOVA followed by Tukey’s multiple comparison test. The values were represented as means±SD. The differences were considered significant when the calculated *P*-value was ˂0.05.

## Results

***Effect of silymarin on WBCs, neutrophil, and lymphocyte count***

ALI significantly increased the WBCs and neutrophil count while it decreased the lymphocytic count in comparison with the normal control group. Silymarin significantly decreased the WBCS but not the neutrophils with a marked increase in lymphocytic count in comparison to the ALI non treated rats (Table 2).

***Effect of silymarin on oxidative stress parameters***

ALI significantly decreased both SOD and CAT activities while increasing the MDA level in the lung tissue compared with the normal control group. Silymarin significantly increased the SOD and CAT activities and decreased the MDA level in the lung tissue in comparison to the ALI induced non treated rats (Table 3).

***Effect of silymarin on Bcl-2 and TGF-β detection by flow cytometry***

**Figure 1:**
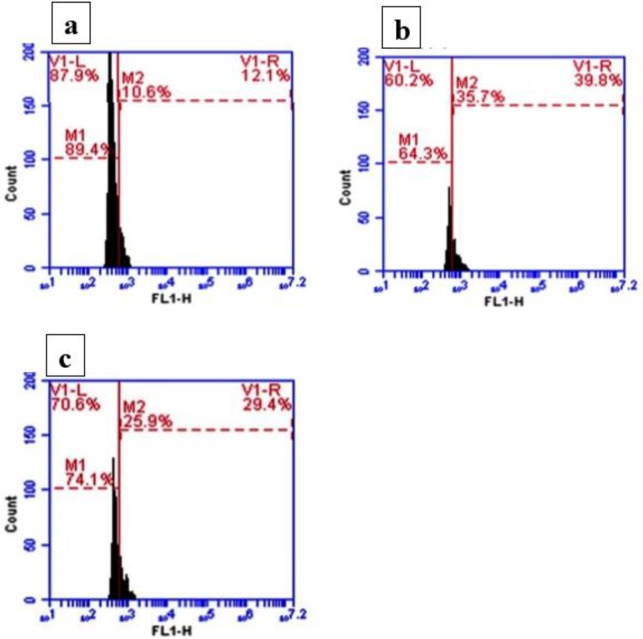
Effect of silymarin on Bcl-2 detection by flow cytometry

The percentage of Bcl-2 protein positive cells for normal lung tissues was 89.49% (Figure 1 a) while this percentage was decreased to 64.3% in ALI induced rats (Figure 1 b). Silymarin was able to increase the percentage of positive cells to 74.9% (Figure 1 c). 

**Figure 2:**
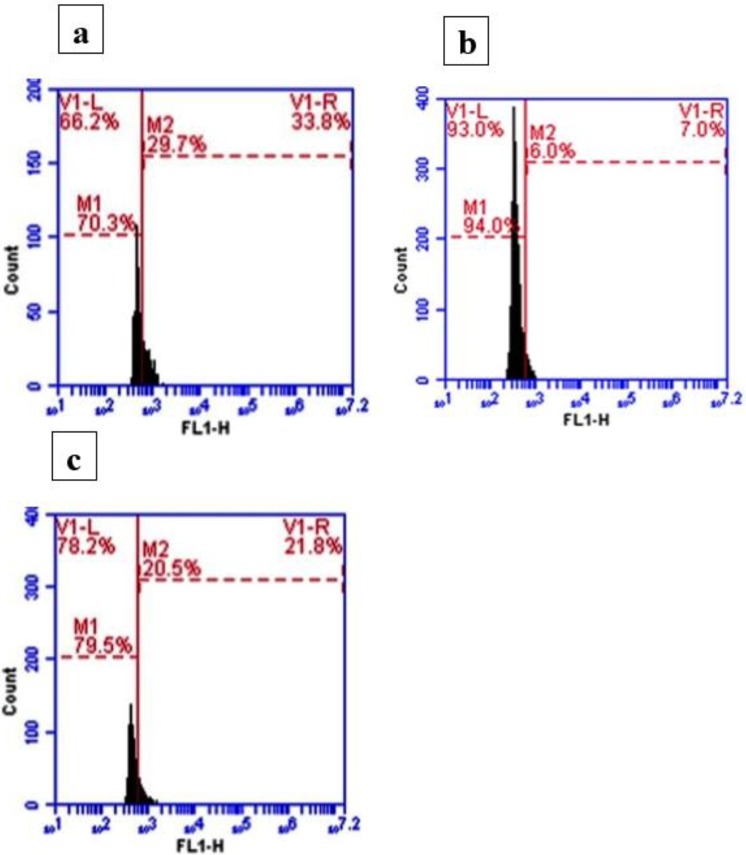
Effect of silymarin on transforming growth factor-beta (TGF-β) detection by flow cytometry

The percentage of TGF-β positive cells in normal control rats was about 70.3% (Figure 2 a), and this percentage was increased to about 94.09% in response to ALI (Figure 2 b). Silymarin treatment showed a detectable decrease in the number of TGF-β positive cells, about 79% of the total cells (Figure 2 c). 

***Effect of silymarin on COX-2, Nrf-2, and HO-1 gene expression***

**Figure 3:**
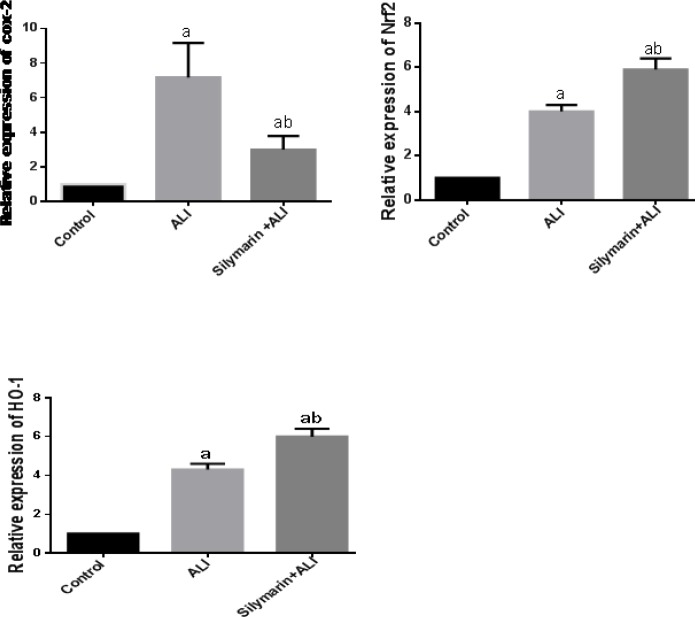
Relative mRNA expression by polymerase chain reaction (PCR) relative to that of the normal control group

ALI was associated with significant increase in mRNA expression of COX-2, Nrf-2, and HO-1 expression in comparison to the normal control group. Silymarin treatment showed significant down-regulation of COX-2 expression; however, Nrf-2 and HO-1 expression were significantly up-regulated by silymarin in comparison to the ALI induced group (Figure 3).

***Histopathological examination***

**Figure 4:**
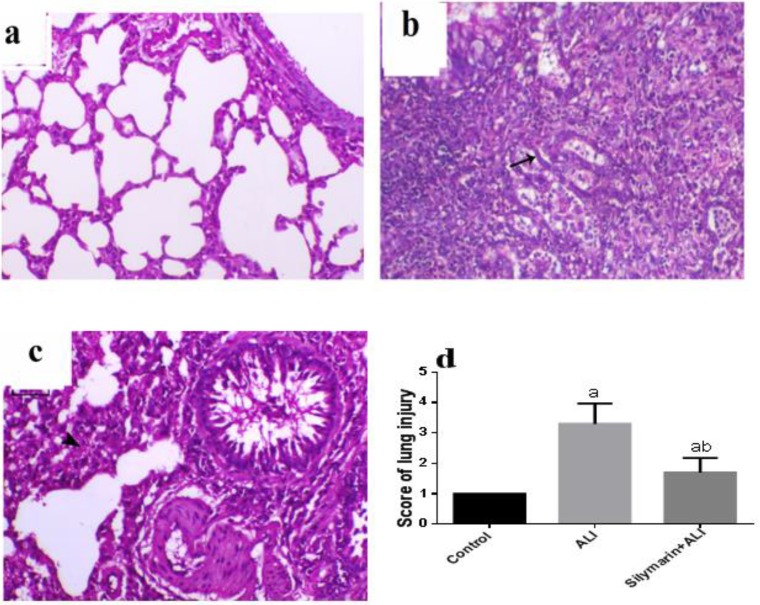
Histopathology of lung tissue

The lungs of control animals showed normal bronchi with normal epithelial lining and normal alveoli separated with thin septa of inter-alveolar connective tissue (Figure 4 a). In contrast, the lungs of ALI animals revealed features of moderate bronchopneumonia, mostly multifocal, and peribronchial pattern. The bronchi demonstrated loss of bronchial lumen patency, epithelial lining degeneration, necrosis, and desquamation. The alveoli showed epithelization of the lining epithelium, which completely filled with foam macrophage and severe fibrin admixed with necrotic tissue. Also, there was marked interstitial connective tissue septal proliferation associated with marked inflammatory cell infiltration (Figure 4b). The animals undergone the same injury and treated with silymarin showed a decrease in the bronchial and alveolar degenerative changes with patent bronchi and alveoli (Figure 4c). Semiquantitative scoring of the lung injury showed significant increase in lung injury in comparison to control rats, however, treatment with silymarin significantly decreased this score compared with ALI rats (Figure 4d).

***Immunohistochemistry of lung tissue***

**Figure 5.**
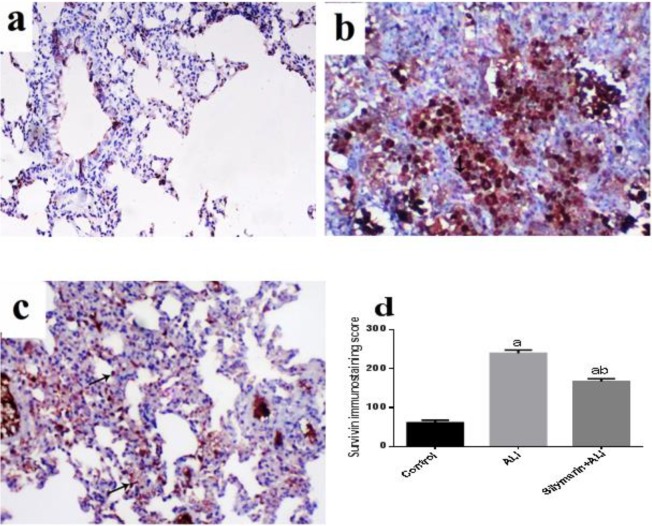
Immunohistochemical staining of lung tissue survivin (magnification X200)

Regarding contra survivin immunostaining, the control lung showed low survivin expression, whereas survivin-positive epithelial cells (nuclear and cytoplasmic) were limited to the bronchial epithelial lining and few alveolar lining cells (Figure 5a). In animal subjected to ALI, the survivin expression was markedly increased either in bronchial or alveolar lining cells. Most of the alveolar cells lined with cuboidal cells showed marked immunoexpression (Figure 5 b). Animals subjected to ALI and treated with silymarin showed similar low survivin expression either within the bronchial or alveolar tissues with statistically significant improvement of immunostaining score (Figures 5 c, d). 

**Figure 6:**
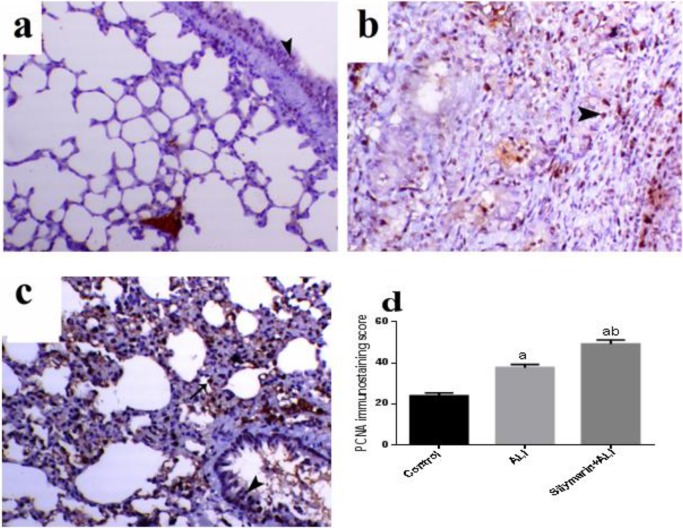
Immunohistochemical staining of lung tissue proliferating cell nuclear antigen (PCNA) (magnification X200)

Concerning PCNA immunolabelling, it was normally expressed within the active dividing cells of bronchial and alveolar lining cells (Figure 6 a). While in the animal undergone ALI, it was fairly increased in bronchial and alveolar tissues, whereas most of the increased expression was within the interstitial tissue mainly within the infiltrated inflammatory cells (Figure 6 b). The animal subjected to ALI and treated with silymarin showed marked increase in the PCNA expression within the bronchial and alveolar epithelial lining compared with normal and ALI rats (Figures 6 c, d).

**Table 1:** List of primer sequences for real-time polymerase chain reaction (PCR)

Gene	Primer forward	Primer reverse
HO-1	**TCACCTTCCCGAGCATCGAC**	**TCACCCTGTGCTTGACCTCG**
Nrf-2	**CATTTGTAGATGACCATGAGTCGC**	**GCTCCATGTCCTGCTGTATGC**
COX-2	**TGTTCCATTTGTGAAGATTCCTGTG**	**TTCTCACTGGCTTATGCCGAA**
GAPDH	**CCAGGGCTGCCTTCTCTTGT**	**CTGTGCCGTTGAACTTGCCG**

**Table 2:** Effect of silymarin on white blood cells (WBCs), neutrophil, and lymphocyte count

Group	WBCs (x10^3^/µl)	Lymphocytes (x10^3^/µl)	Neutrophils (x10^3^/µl)
Control	14.8 ±2.18	10.65±1.95	2.54±0.39
ALI	23.08±6.43^a^	6.48±0.69^ a ^	4.44±0.51^ a^
Silymarin+ALI	16.3 ±4.87^b^	12.1 ±2.44 ^b^	4.03±0.46^ a^

Values are representation of 10 values as means±SD. Results are considered significantly different when *P*<0.05. aSignificant difference compared with the control group, b significant difference compared with the ALI group. ALI: acute lung injury; WBCs: white blood cells

**Table 3:** Effect of silymarin on oxidative stress parameters

Groups	SOD(U/g tissue)	CAT (U/g tissue)	MDA (nmol/g tissue)
Control	1358±25	4.56±0.12	29.53±0.21
ALI	1099±38^ a^	3.3±0.26^ a^	34.82±0.28^ a^
Silymarin+ALI	1273±70^ ab^	4.51±0.14^b^	32.16±0.45^ ab^

Values are representation of 10 values as means±SD. Results are considered significantly different when *P*<0.05. aSignificant difference compared with the control group, b significant difference compared with the ALI group. ALI: acute lung injury, SOD: superoxide dismutase, CAT: catalase; MDA: malondialdehyde

a: Control group: shows the normal percentage of positive cells for Bcl-2 protein. b: ALI induced group: shows increased percentage of positive cells for Bcl-2 protein. c: Silymarin+ALI group: shows a decrease in positive cells for Bcl-2 protein compared with the ALI group. ALI: acute lung injury

a: Control group: shows high percentage of TGF-β positive cells. b: ALI induced group: shows decreased percentage of TGF-β positive cells compared with control rats. c: Silymarin +ALI group: shows increased percentage of TGF-β positive cells compared with ALI but less than control rats. ALI: acute lung injury

Values are representation of 10 values as means±SD. Results are considered significantly different when *P*<0.05. aSignificant difference compared with control group, bsignificant difference compared with the ALI group. ALI: acute lung injury

Hematoxylin and eosin evaluation of rat lung (magnification x200): (a) Lung of normal animal showing normal alveolar and bronchial structures. (b) Lung of ALI animal showing epithelialization of the alveolar lining (arrow) associated with pneumonia and marked inflammatory cell infiltration either intra-alveolar or interstitial and heavily fibrous exudate deposition. (c) The lung of animal undergone lung injury and treated with silymarin showing moderately decreased interstitial thickening (arrowhead) and bronchial epithelium necrosis. (d) Semiquantitative scoring of lung injury. Values are representation of 10 values as means±SD. Results are considered significantly different when *P*<0.05. aSignificant difference compared with the control group, b significant difference compared with the ALI group. ALI: acute lung injury

(a) Lung of normal rat showing normal PCNA immunolabeling within the bronchial (arrowhead) and alveolar tissues. (b) Lung of ALI rats showing relatively increased PCNA immunostaining within the alveolar and bronchial epithelial cells (arrowheads indicate positive nuclear expression) while the interstitial inflammatory cells reveal higher expression. (c) Lung of animal undergone lung injury and treated with silymarin showing increased PCNA immunostaining within the alveolar (arrow) and bronchial epithelial cells. Semiquantitative scoring of lung PCNA in which values are represented as means± SD. Results are considered significantly different when *P*<0.05. a Significant difference compared with control group, b significant difference compared with the ALI group. ALI: acute lung injury

## Discussion

To date, ARDS is still the leading cause of respiratory failure in critically ill patients in the ICU with high mortality rate. Gastric acid aspiration is a major direct cause of severe ARDS (9). The pulmonary protective effect of silymarin was proven against paraquat-induced lung injury (7) but not evaluated in HCl induced ALI. So, this study aimed to evaluate the protective activity of silymarin in acid aspiration induced lung injury. In the current study, intra-tracheal injection of HCl caused an inflammatory response in the form of leukocytosis with increased neutrophil count, up-regulation of COX-2 mRNA expression, and inflammatory cell infiltration of the lung tissue with marked histopathological injury. 

Regarding the leukocyte count in the present study, acid aspiration caused leukocytosis with increased neutrophil count. This result was previously reported in the literature (23). Neutrophil recruitment to the lung is mediated primarily by interleukin-8 that produces a lot of chemokines, cytokines, and intracellular oxidative stress resulting in pulmonary edema (24). Acid aspiration in this study decreased the blood lymphocytic count compared with the control rats, and this result is in accordance with Setzer and his colleagues (23). This result reflects the severity of lung injury as it was reported that 54% of 144 patients with severe ARDS showed lymphopenia (25).

Silymarin treatment normalized leukocyte count as the first sign of inflammation regression, which is in accordance with previously reported data (26).

HCl aspiration in the present study up-regulated COX-2 mRNA, which is consistent with a previous study (27). The inducible COX-2 is an inflammatory marker, which when blocked by parenteral celecoxib can attenuate the inflammatory response and oxygenation impairment after HCl aspiration (28). COX-2 is expressed in resident inflammatory cells in the lung and pulmonary endothelium and epithelium and its induction leads to the increased expression of prostanoids that play key roles in modulating inflammation in the lung (29). Silymarin in this study significantly decreased COX-2 mRNA confirming its anti-inflammatory activity that was previously reported (30). Silymarin was reported to decrease the COX-2 expression in different tissues (31, 32). 

In the current study, the histomorphological signs of lung cell injury have confirmed the severity of inflammatory changes of the lung in response to HCl aspiration as there was marked inflammatory cell infiltration, edema, alveolar wall degeneration, and necrosis as previously reported (32). In accordance with the previously published results, silymarin preserved tissue morphology and reversed the inflammatory lung changes (33).

In the present study, acid aspiration results in marked oxidative stress with increased lung tissue lipid peroxidation end product MDA level, Nrf-2 and HO-1 mRNA expression with decreased SOD, and CAT activities. Our results are in accordance with the previously published data (34, 35). SOD and CAT are well-known antioxidant enzymes in the detoxification of superoxide anion radical (^*^O_2_) and hydrogen peroxide (H_2_O_2_) (36). Decreased SOD and CAT activities lead to accumulation of ^*^O_2 _and H2O2. The reaction of accumulated ^*^O_2 _with membrane lipids resulted in lipid peroxidation with loss of cellular components (37). These oxidative stress changes may explain the injurious effect of HCl on the investigated lungs.

Nrf-2 is a transcription factor with a key role in cytoprotective gene expression. It regulates critical antioxidant and stress-responsive genes and is closely related to the pathogenesis of pulmonary disorders, including ALI (38). The inducible HO-1 catalyzes the rate-limiting step in the catabolism of the pro-oxidant heme to carbon monoxide, biliverdin, and free iron (39). HO-1 has both antioxidant and anti-inflammatory effects, as biliverdin can be reduced to the antioxidant bilirubin, and carbon monoxide has anti-inflammatory and anti-apoptotic effects (40). Moreover, the vasodilator effect of CO is of important role in regulating basal and constrictor-induced vascular tone (41). HO-1 is arguably the most well-known of all Nrf-2-regulated genes however; HO-1 is regulated by multiple mechanisms in addition to Nrf-2 (41). The nrf-2/ho-1 pathway is stimulated and up-regulated in various forms of ALI (42, 43) as a part of an adaptive response to oxidative stress (44). 

In the current study, silymarin exhibited a powerful antioxidant activity as it significantly increased SOD and CAT activities with increased HO-1 and Nrf-2 mRNA expression together with significant decrease in MDA level. Silymarin was reported to prevent sepsis-induced lung and brain (33), carbon tetrachloride (CCL4)-induced liver (45), burn-induced oxidative skin (46), and doxorubicin-induced cardiac (47) injuries via its antioxidant activity. Silymarin was reported to activate the Nrf-2/ HO-1 pathway in different tissues (48). HO-1 up-regulation results in an increase in the activity of SOD, CAT, and endothelial nitric oxide synthase (eNOS) with a concomitant increase in endothelial relaxation and a decrease in ^*^O_2_ level (49) with consequently decreased lipid peroxidation (37). So we can consider that induction of Nrf-2/HO-1 pathway is the main mechanism of silymarin protection against HCl induced ALI. 

Bcl-2 is an anti-apoptotic protein that prevents the translocation of pro-apoptotic protein Bax to the mitochondria (50). The present study revealed a significant decrease in the level of anti-apoptotic protein Bcl-2 in response to HCl aspiration. Bcl-2 is down-regulated in different types of ALI like LPS (51), and prolonged hyperbaric hyperoxia (52) induced ALI. Silymarin treatment significantly increased Bcl-2 level compared with the non-treated ALI group which confirms its anti-apoptotic activity that was previously reported (53, 54). There is interaction among oxidative, inflammatory, and apoptotic pathways and there is difficulty in determining whether the relationship between these pathways is a cause or a consequence of one another (55).

TGF-β is a cytokine that plays a critical role in tissue injury resolution of many organs, including lung (56). TGF-β is the most thoroughly evaluated during the late phases of tissue repair following ALI, where it plays a critical role in the development of lung fibrosis (57). Consistent with the previously published data, HCl in the present work increased the TGF-β protein, which was decreased by the effect of silymarin treatment nearly to its normal level, which confirmed the anti-fibrotic activity that was previously reported for silymarin (58, 59).

Survivin is a member of the apoptosis inhibitor family that regulates cell division (6). It was reported that survivin was involved and up-regulated in ALI induced by bleomycin and was considered as the key mediator of cytoprotection (6). The result of the present study revealed increased survivin immunostaining in response to HCl aspiration, which was significantly prevented by silymarin treatment. It was reported that survivin increased after LPS-induced ALI in mice and its level was decreased with damage resolution (60). So, we can hypothesize that the anti-inflammatory and antioxidant activities of silymarin lead to resolution of ALI with subsequent decrease in lung tissue survivin immunostaining.

PCNA is an essential DNA replication and repair protein that increases significantly in proliferating cells (61) as a marker of proliferation and DNA damage repair (62). In the present study, HCl aspiration significantly increased the PCNA immunostaining, which means that HCl caused DNA damage to alveolar epithelial cells. The increased PCNA was associated with the proliferative phase in ALI, which was induced by certain cytokines and growth factors (63). PCNA overexpression was previously reported in bleomycin (61), asbestos (64), and nitrogen mustard (65) induced ALI. In the present study, silymarin treatment increased PCNA expression more than that which occurred in both control and ALI groups, reflecting the proliferative capacity of silymarin. A similar result reported that silymarin treatment increased the number of proliferating (PCNA-positive) cells in mice liver intoxicated by fumonisin B1 and this was a part of its protective effect (66). Limitations of this study: firstly, though the studied dose of silymarin was chosen according to our preliminary study and based on previous studies (7, 20), it is worthy to note that the result of the current study is limited due to using a single dose of silymarin, and further evaluation of multiple doses is needed to confirm the present conclusion. Secondly, this study lacks the evaluation of the target proteins of the expressed genes by the Western blot method for confirmation of the studded pathway.

## Conclusion

This study concluded that silymarin has ameliorative effect against HCL induced ALI via its modulation of Nrf-2/HO-1 pathway with subsequent anti-oxidant, anti-inflammatory, anti-apoptotic, proliferative and regenerative effects

## Funding Source

This research did not receive any specific grant from funding agencies in the public, commercial, or not-for-profit sectors.

## Conflicts of Interest

The authors declare that there are no conflicts of interest regarding the publication of this paper.
